# Accelerometry-Based External Load Indicators in Sport: Too Many Options, Same Practical Outcome?

**DOI:** 10.3390/ijerph16245101

**Published:** 2019-12-13

**Authors:** Carlos D. Gómez-Carmona, José Pino-Ortega, Braulio Sánchez-Ureña, Sergio J. Ibáñez, Daniel Rojas-Valverde

**Affiliations:** 1 Optimization of Training and Sport Performance Research Group (GOERD), Sport Science Faculty, University of Extremadura, 10005 Caceres, Spain; sibanez@unex.es; 2 Department of Physical Activity and Sport, International Excellence Campus “Mare Nostrum”, Sport Science Faculty, Universidad de Murcia, San Javier, 30720 Murcia, Spain; 3 Program of Exercise Science and Health (PROCESA), School of Human Movement Science and Quality of Life, Universidad Nacional, Heredia 86-3000, Costa Rica; braulio.sanchez.urena@una.cr; 4 Research Center of Sport and Health Diagnosis (CIDISAD), School of Human Movement Science and Quality of Life, Universidad Nacional, Heredia 86-3000, Costa Rica; drojasv@una.cr; 5 Group in Updates for Sport Training and Physical Conditioning (GAEDAF), Sport Science Faculty, University of Extremadura, 10005 Caceres, Spain

**Keywords:** accelerometers, load indexes, team sports, sport technology, monitoring

## Abstract

With the development of new microsensor technology to assess load in sports, some indicators of external load through accelerometry-based data have been created by sport technology companies. Thus, the study aim was to analyze the agreement between different accelerometry-based external load indicators (ABELIs) available in sport science. A U-16 male soccer team was assessed during three official matches, divided by periods, to obtain 3-D accelerometry data (x, y and z axes). An average of 1,420,000 data points was analyzed per axis per player. The ABELIs were calculated using this information, and the agreement between them was explored. The following ABELIs were considered after a literature review: AcelT, Player Load_RT_, PlayerLoad^TM^, Impulse Load, Player Load_RE_ and Total Load. In order to compare ABELIs, two analyses were performed using: (1) absolute data; and (2) normalized and centered data (Z-scores). In absolute and centered data, very large to nearly perfect correlations (1st period: *r* > 0.803, *p* > 0.01; 2nd period: *r* > 0.919; *p* > 0.01) were found. Instead, very large differences were found in absolute values (bias = −579,226.6 to 285,931.1; *t* = −224.66 to 213.91, *p* < 0.01), and no differences in scaled and centered values (bias = 0; *t* = 1; *p* = 1). In conclusion, considering the different output (magnitude and units) among ABELIs, the standardization of a universal index to calculate accelerometer load is needed in order to make possible between-study comparison.

## 1. Introduction

Currently, the monitoring of internal and external loads is very important for understanding the demands placed on players, and for designing specific training loads, tactical strategies, injury prevention programs and recovery methods in sport [1,2,3,4,5]. Thanks to technological advances, new devices known as electronic performance tracking systems (EPTS) have been developed for this purpose and are able to record up to a thousand data points per second [6]. These devices are composed of different sensors such as accelerometers, gyroscopes, magnetometers, indoor and outdoor tracking sensors and external sensors that communicate with them through Ant+, Bluetooth or Wi-Fi technology (heart rate telemetry, muscle oxygen saturation, internal temperature, power, cadence, among others) with excellent accuracy and reliability [7,8,9,10,11,12].

One of these sensors is the accelerometer. Accelerometers were first introduced in sports science in the beginning of the 21st century thanks to the Project 2.5 “Technology of Communication to Athletes Monitoring” carried out by the Australian Centre of Microtechnological Research for designing a unique nonintrusive device for sport monitoring in real time [13]. Since its appearance, there has been enormous technological, technical and methodological development in the use of accelerometry to quantify external workload in sports [14,15,16]. The sensitivity and precision of accelerometry-based variables are higher compared to other tracking systems that underestimate load demands. This phenomenon is due to the fact that static high-intensity actions without covering ground (jumps, collisions, falls, tackles, etc.) cannot be recorded by time–motion systems, but can be measured with high accuracy by accelerometers [6,17].

A large quantity of variables has been developed using data recorded by this sensor. The analysis of this information has tended to be more complex because the different companies use different algorithms to classify the actions and this limits comparability among studies [18]. The most used variable is PlayerLoad^TM^, designed by Catapult Sports company [11]. This variable was created to quantify the total load players are exposed to and is obtained from the acceleration in the three axes recorded by the accelerometers, measured in arbitrary units (a.u.), with high reliability and validity [19,20,21]. Other variables have been created with the same purpose, such as vectorial sum of acceleration (a(t)) [22,23,24] used by almost all the companies, Player Load developed by the company RealTrack Systems (Player Load_RT_) [25], Impulse Load created by Zephyr^TM^ [26], New Body Load by GPS Sports [27], Player Load by the manufacturer ZXY SportTracking (Player Load_RE_) [17] and Total Load by StatSports [28]. All the companies use accelerometer data from the vertical, horizontal and medio-lateral planes but the calculations made to extract the final external workload are quite different, complicating comparison among them.

In this respect, two important aspects of accelerometry-based external load indicators (ABELIs) have been considered: (a) the difference among the algorithms used to calculate the ABELIs and the resulting magnitude and unit variability among them hinder their practical application by team staff and sport scientists due to the different devices used [29]; and (b) different technical aspects such as variety of sampling frequencies, chip sets, filtering methods and data-processing algorithms also influence interdevice comparison among ABELIs. Due to these differences in data processing between brands/models of EPTS devices, clubs currently cannot compare data between ABELIs and cannot recalculate the accelerometer-load indexes according to their preferences for players that are away with the national team, where the external load may have been captured with an accelerometer device different from the one the club uses [18].

In addition, if total variables of ABELIs are used for load management, only the total volume of the session is considered for further analysis. Thus, due to the physiological particularities of team sports, especially in indoor conditions where high-intensity actions with short recovery periods are performed [1,2,30,31], ABELIs should be considered related to playing time to be more representative of the general effort involved in the session/competition. This aspect includes the intensity of intermittent efforts in the final analysis and seems to provide more precise information on demands. Besides, it will make comparison possible independently of the playing time [5,31,32].

Despite the information presented above, there is a lack of research on the relationship and differences between accelerometry-based external load indicators (ABELIs) available in sport science. Researchers, coaches and athletes have in ABELIs a fundamental tool to assess players’ total external workloads during training and competition [33]. Based on this information practitioners could develop new accelerometry-based protocols of training prescriptions, competition strategies, periodization, training cycles and return to play protocols, among other exercise prescription programs; all based on individualized player data [5,34]. Despite increasing evidence on external load variables during competition and training in diverse sports, the use of different ABELIs by researchers does not allow the application of this information by a different company user. New evidence is needed about the relation among all these ABELIs because it is hypothesized that as all algorithm indexes come from the raw data of triaxial accelerometers, relationships and no differences will be found when variables are normalized. Therefore, the aim of this study was to analyze the agreement among the different accelerometry-based load indicators available in sport science.

## 2. Materials and Methods

### 2.1. Sample and Participants

A total of 1,420,000 data points were analyzed per axis, derived from the assessment of both periods (40.60 ± 0.40 min) of three official U-16 soccer matches in order to obtain accelerometry data on three axes (x, y and z). The data were analyzed by period due to the differences reported in previous soccer studies in physical performance indicators and between substituted and nonsubstituted players [35]. All players, both substitutes and those who played the entire match, were included in the analysis. Only the activity when the players were participating in the game was considered.

For data collection, eighteen under-16 male soccer players (age: 15.6 ± 0.8 years; body mass: 64.5 ± 5.2 kg; height: 172.3 ± 11.2 cm) participated voluntarily in the present study. All the players taking part were from a soccer club that played in the Spanish First Regional Division and met the following inclusion and exclusion criteria: (a) not presenting any musculoskeletal injury or health problem that impeded their participation in competition games; (b) having received 3 months of high-level monitoring by electronic performance tracking systems (EPTS) both in training and official games [36]; (c) goalkeepers did not take part in the final sample due to the physical load differences with all the playing positions in the field [37].

Both technical staff and players were previously informed about the investigation details and signed an informed consent. As all players were minors, consent was signed by their legal guardians. The study was performed based on the ethical guidelines of the Declaration of Helsinki (2013) and approved by the Bioethics Committee of the University (registration number 2061/2018). The club authorized all action protocols.

### 2.2. Instruments and Procedures

Three official matches from one under-16 Spanish male First Regional Division team were recorded using inertial measurement units WIMU PRO^TM^ (RealTrack Systems, Almeria, Spain). These devices contain four 3D accelerometers, as well as other sensors (three 3D gyroscopes with 8000°/s full-scale output range, a 3D magnetometer, a 10-Hz global positioning system, a 20-Hz ultra-wide band), that detect and measure movement using a micro-electromechanical system with an adjustable sampling frequency from 10 to 1000 Hz. The full-scale output ranges of the four 3D accelerometers are ±16, ±16, ±32 and ±400 g. Furthermore, each device has its own GHz microprocessor, 8 GB flash memory and a highspeed USB interface, to record, store and upload data. The device is powered by an internal battery with 4 h of life. The dimensions of each WIMU PRO^TM^ were 81 mm × 45 mm × 16 mm, and the weight was 70 g.

An autocalibration process was performed before the data acquisition, following the manufacturer’s recommendations: (a) to switch on the device and not move it for 10 to 15 s; (b) to leave it on a flat zone; and (c) to not have magnetic objects around it. Even so, to ensure the perfect functioning of the accelerometry sensors, a manual calibration process was carried out where the device must be placed static on its six faces for 10 s where the values should be 1 ± 0.01 G. To obtain a better signal, the accelerometer company introduced some data filtration processes related to the different sampling frequencies and output range, these filtration stages were applied before “raw data” were available for the user. In this case, the filtration processes were performed at three levels: (1) accelerometer manufacturer; (2) inertial device chipset; and (3) software filtration. These filtering processes are not controlled by the user and they are applied before “raw data” is available. With this calibration process, the accelerometers in the inertial device have obtained very satisfactory results for static and dynamic reliability in laboratory and real-context field conditions [38].

The inertial measurement units (IMUs) were used to record the players’ accelerometer load data during the official games. All the players wore a special neoprene vest and the IMU was attached at the T2–T4 level in the medial line between scapulae prior to the warm-up for each match. In the present research, the fusion of the data from the four accelerometers per axis was performed based on the redundancy principle to increase reliability. The sampling frequency used was 100 Hz. The playing time of each player was recorded in real time by the software SVIVO^TM^. At the end of each recording, data from the inertial devices and time selection were imported by SPRO^TM^ software to download the accelerometer data per axis (RealTrack Systems, Almeria, Spain).

### 2.3. Accelerometry-Based External Load Indicators (ABELI)

For the present investigation, an online database review was carried out on the different accelerometry-based external load indicators (ABELIs) available in sport sciences. Then, each of these variables was described, identifying the description of the variable, the measurement units, the developing company and the formula for calculating each of them, that is based on the acceleration raw data recorded in each axis during movement. All ABELIs are calculated from the vector sum of the acceleration in the three planes of movement accumulated during the official match, resulting from the sum of the accelerations over a time period (e.g., 40 min). Three axes of movement, x, y and z, refer to the vertical, medio-lateral and anterior–posterior acceleration, respectively, in the following equations.

- a(t) (developing companies: ActiGraph LLC and GENEActiv; units: g force, *g*) [22,23]: Square root of the sum of the accelerations in the three accelerometer orthogonal axes (x, y and z), measuring the combination of gravity and changes in vertical, medio-lateral and anterior–posterior motions of a body segment to which the accelerometer is attached (Equation (1)).
  (1) $$\overset{\;}{\underset{\;}{\sum }}\sqrt{\left(x^{2}+y^{2}+z^{2}\right)}$$
- Player Load_RT_ (developing company: RealTrack Systems; units: arbitrary units, a.u.) [25]: Vector sum of the four accelerometer data points in its three axes of movement (vertical, anteroposterior and lateral). It is represented in arbitrary units (a.u.) and is calculated from the following equation where *PL_RT_* is the player load calculated in the current moment; *X_n_*, *Y_n_* and *Z_n_* are the values of BodyX, BodyY and BodyZ in the current moment; and *X_n–1_*, *Y_n–1_* and *Z_n–1_* are the values of BodyX, BodyY and BodyZ in the previous moment. Then, the sum of *PL_RT_* during the session is calculated and multiplied by 0.01 as a scale factor (Equation (2)).
  (2) $$PL_{RT}=\sqrt{\frac{{\left(X_{n}-X_{n-1}\right)}^{2}+\;{\left(Y_{n}-Y_{n-1}\right)}^{2}+\;{\left(Z_{n}-Z_{n-1}\right)}^{2}}{100}\;}\;\;PL\;acummulated\;=\overset{m}{\underset{n=0}{\sum }}PL_{RT}\;\times \;0.01\;$$
- PlayerLoad^TM^ (developing company: Catapult Sports; units: arbitrary units, a.u.) [11]: Vector sum of the changes in acceleration in the anterior–posterior (forward) medio-lateral (side) and vertical (up) planes (Equation (3)).
  (3) $$\overset{\;}{\underset{\;}{\sum }}\sqrt{\frac{{\left(fwd_{t=i+1}-\;fwd_{t=i}\right)}^{2}+{\left(side_{t=i+1}-\;side_{t=i}\right)}^{2}+{\left(up_{t=i+1}-\;up_{t=i}\right)}^{2}}{100}}\;$$
- Impulse Load (developing company: Zephyr^TM^; units: newtons per second, N/s) [26]: A cumulative sum of the forces in *x* = g forces in the medio-lateral (“side-to-side”) plane, *y* = g forces in the anterior–posterior (“forwards and backwards”) plane, and *z* = g forces in the vertical (“up and down”) planes of movement. This is then scaled by gravity (Equation (4)).
  (4) $$\overset{n}{\underset{s=1}{\sum }}\frac{\sqrt{x_{s}^{2}+\;y_{s}^{2}+z_{s}^{2}}\;}{9.8067}\;$$
- Player Load_RE_ (developing company: ZXY SportTracking; units: arbitrary units, a.u.) [17]: The player load is calculated and presented as a downscaled (i.e., divided by 800) value of the square sum of the accelerometer values for the respective axes (x, y, and z). Thus, the load value is the downscaled square of the player’s absolute acceleration. The downscaling was used for practical reasons (Equation (5)).
  (5) $$\overset{\;}{\underset{\;}{\sum }}\frac{\left(x^{2}+y^{2}+z^{2}\right)}{800}\;$$
- Total Load (developing company: StatSports; units: arbitrary units, a.u.) [28]: Total accumulated accelerations of the player based on accelerometer data, where *aca* is acceleration along the anterior–posterior axis, *acl* is acceleration along the lateral axis and *acv* is acceleration along the vertical axis, *i* is current time and *t* is time. This is then scaled by 1000 (Equation (6)).
  (6) $$\overset{\;}{\underset{\;}{\sum }}\frac{\sqrt{{\left(aca_{t=i+1}-\;aca_{t=i}\right)}^{2}+{\left(acl_{t=i+1}-\;acl_{t=i}\right)}^{2}+{\left(acv_{t=i+1}-\;acv_{t=i}\right)}^{2}}}{1000}\;$$

### 2.4. Statistical Analysis

All external load estimation formulas were calculated using a special digital sheet (Excel, Microsoft, Redmond, WA, USA) based on the accelerometer raw data (x, y and z axes) given by the IMU. Calculation outcomes were presented in means, lower and upper limits and standard deviation. The Kolmogorov–Smirnov test was used to confirm the normality of the data, verifying the feasibility of using parametric inference. Two agreement and correlation analysis between ABELIs was performed using: (1) absolute data; and (2) scaled and centered (Z-score) data. Z-scored data were used in order to standardize the units and magnitudes of each variable [39], this data transformation is made to have uniform scale so they can be analyzed, the purpose is to equalized the size, magnitude and variability of the input variables [40].

Agreement among the different ABELI was analyzed using the most common statistical tests following previous study principles [41,42]. The correlation and comparison based tests used to report agreement were: (1) r-Pearson to confirm and explore lineal correlation among ABELI; (2) intraclass correlation coefficient (ICC) and a 95% CI; (3) bias was explored using the Bland and Altman method [43]; (4) mean differences among variables were analyzed using *t*-tests.

The magnitude of the differences was qualitatively interpreted using Cohen’s *d* (*d*) as follows: >0.2 trivial; 0.2–0.49 small; 0.5–0.79 moderate and >0.8 large [44]. ICC was interpreted following previously proposed ranks as: poor (0), trivial (0.01–0.02), regular (0.21–0.4), moderate (0.41–0.6), substantial (0.61–0.8), and almost perfect (0.81–1) [45]. The Pearson correlation coefficient was interpreted as trivial (r^2^ < 0.1), small (0.1 < r^2^ < 0.3), moderate (0.3 < r^2^ < 0.5), large (0.5 < r^2^ < 0.7), very large (0.7 < r^2^ < 0.9), nearly perfect (r^2^ > 0.9) and perfect (r^2^ = 1) [46]. Statistical analyses were performed using IBM SPSS Statistics (version 24, IBM Corporation, Armonk, NY, USA). Statistical differences were considered if *p* < 0.05.

## 3. Results

### 3.1. Descriptive Analysis

Table 1 shows the descriptive analysis of the accelerometry-based external load indicators (ABELI) obtained as the average of three U-16 official soccer matches, divided by periods of match play. A great variability of data was found among ABELI, where the lowest values were obtained by Player Load by RealTrack Systems (PL_RT_) and Total Load by StatSports (TL) and the highest values was obtained by total acceleration, a(t).

### 3.2. Agreement of ABELIs’ Measures

The agreement of measures of accelerometry-based external load indicators (ABELI) in young soccer players, divided by periods, is shown in Table 2 (absolute data) and Table 3 (centered and scaled data). Table 2 shows that a very large to nearly perfect correlation was found between ABELIs in the first and second period by Pearson’s correlation coefficient (1st period: *r* > 0.803, *p* > 0.01; 2nd period: *r* > 0.919, *p* > 0.01) but the intraclass correlation coefficient was trivial to substantial between indexes (1st period: ICC = 0.003 to 0.729, 95% CI = −0.601 to 0.925; 2nd period: ICC = 0.001 to 0.974, 95% CI = −0.602 to 0.994). Besides, in comparison analysis, very large differences were obtained in Bland–Altman (bias = −579,226.6 to 285,931.1), Student’s *t* for independent samples (*t* = −224.66 to 213.91; *p* < 0.01) and large effect size for partial omega squared (ω_p_² = 0.28 to 1.00).

On the other hand, when data were scaled and centered as shown in Table 3, a very large to nearly perfect correlation was found among ABELIs in the first and second period, both in Pearson correlation coefficient (1st period: *r* > 0.803, *p* > 0.01; 2nd period: *r* > 0.919, *p* > 0.01) and intraclass correlation coefficient (1st period: ICC > 0.803, 95% CI = 0.394 to 1.00; 2nd period: ICC > 0.918, 95% CI = 0.707 to 1.00). Besides, in comparison analysis, no differences in Bland–Altman (bias = 0), Student’s *t* for independent samples (*t* = 1; *p* = 1) and trivial effect size for partial omega squared were obtained (ω_p_² = 0). Perfect correlations were found in both periods between a(t) vs. impulse load by Zephyr^TM^ (IL) and PL_RT vs._ PlayerLoad^TM^ by Catapult (PL^TM^).

## 4. Discussion

Thanks to technological advances in the sport science area, inertial devices with different sensors such as accelerometers have been utilized for load quantification in individual and team sports. Although all indexes provide information from the acceleration of the three axes of movement, as each company has developed an independent ABELI for workload monitoring, a comparison cannot be made among indexes. Therefore, the aim of the present research was to analyze the agreement among the different accelerometry-based load indicators available in sport science. The main results in this research found very large to nearly perfect correlations in absolute and scaled and centered data (1st period: *r* > 0.803, *p* > 0.01; 2nd period: *r* > 0.919, *p* > 0.01). Instead, very large differences were found in absolute values (bias = −579,226.6 to 285,931.1; *t* = −224.66 to 213.91; *p* < 0.01), and no differences were found in scaled and centered values (bias = 0; *t* = 1; *p* = 1).

The absolute data obtained in the present study were similar to those reported in recent published research. Regarding studies exploring the locomotor demands in young soccer players, the most used index was PL^TM^ [1]. In the present study a PL^TM^ of 12.8 a.u./min was found, while other published data reported 10.2 a.u./min in young male soccer players [47], 11.5 a.u./min in elite male soccer players [48], and 10.3–10.9 a.u./min in consecutive matches in elite female players [49] using the same variable. These differences between the values obtained in literature and the present results could be due to the lack of tactical synchronization in U-16 with respect to U-18 and senior players that cause more displacements [50] and shorter periods of time (40 vs. 45 min) that produce less fatigue [51]. The obtained PL^RT^ was 1.3 a.u./min, while in official youth soccer games, recent evidence reported 1.8 a.u./min [52], and 1.4 a.u./min was reported in semiprofessional male soccer players [25]. The level of the soccer team/opponents and the match outcome also may be an influential factor [53,54].

In respect to IL, the results obtained in this study (656.5 N/min) suggest higher values compared to those obtained in similar research on female soccer players (447.1 N/min) [26]. Female soccer teams covered shorter distances with less intensity than male soccer teams. Additionally, TL obtained was 1.3 a.u./min, similar to other data extracted from youth soccer matches and training (1.4 a.u./min) [28]. It should be considered that the results extracted in this study were obtained from a youth soccer team and this may analyzed with caution when these data are extrapolated to other competition categories due to the clear physical and technical differences between both competition groups [55].

Regarding a(t), a total of 6438.1 *g*/min was found in the present study, but higher cumulative data of 8040 *g*/min has been recorded in other studies in a specific training soccer circuit [38]; therefore there are not enough references using a(t) in soccer play. Finally, it is difficult to compare the data on PL_RE_ obtained in this research because the ZXY SportTracking device used fits the IMU on the waist, and other systems fit the units at the T2−T4 level between the scapulae; due to this fact, the values obtained are lower than those published previously [17] because the workload resulting from lumbar segments tends to be higher than those assessed in thoracic segments [20,38,56,57]. In this sense, previous research realized by Nedergaard et al. [56] reported that a body-worn accelerometer only measures the acceleration of the segment that it is attached to, therefore, worse agreements could be expected with the other ABELIs due to the different measurement location.

The high correlation and agreement found between all ABELIs is because all the indexes are calculated using the accumulated change in acceleration of the three axes of movement, with the same units and at the same level (scapulae). However, values between companies are different due to the applied algorithms, units and magnitudes. For this reason, differences between absolute data were found between ABELIs. Therefore, it is important to analyze the validity and reliability of device accelerometers, and companies need to explain how many filtering processes have been carried out before the user downloads the “raw data” for comparing the implementation of all ABELIs in all accelerometers or inertial devices [38]. Despite that there was high agreement between these indicators, future studies could explore the sensitivity of the ABELIs to detect changes in physiological or mechanical loads and the ability to detect the most demanding high intensity tasks or risk of overuse injuries among other applications.

While the results of this study have provided information about the agreement and the differences between accelerometer-based external load indicators during three U-16 soccer matches, some limitations to the study must be acknowledged. Although the sample used for this study was reduced (13 players per match in three official games, divided by match periods), an average of 1,420,000 data points in each axis of movement per player was generated to calculate all the ABELIs using the accelerometer data at a sampling frequency of 100 Hz. The limitations of applying 3D accelerometer-based load indicators include summation of accelerations that mask the directional profile (medio-lateral or antero-posterior or vertical) and application of the square and then square root to data that negates orientation of acceleration (medial or lateral, anterior or posterior, take-off or landing). Therefore, to solve this problem, new variables were generated to analyze the individual axis contributions in accumulated accelerometry-based workload [20,58].

Additionally, a limitation encountered in the present research is the difficulty to access the ABELI formulas of EPTS companies, in addition to the lack of information in the sport science area related to sampling rates, chip sets, filtering methods and data-processing algorithms, which makes it impossible to compare devices from the accelerometer raw data. Besides, specific IMU devices have been used in the present research, so, in order to compare ABELIs with other companies, not only should we have the formula to calculate it, but also the specific sampling frequency, chip sets, filtering methods and fusion of accelerometer data based on the redundancy principle that is used by each company. Future research should explore how this different collecting and processing of data could affect the ABELI’s outcome. It should be taken into account that each IMU manufacturer usually applies some filtration processes before the accelerometer “raw data” is available for users, and these filtration stages and filters could differ between companies.

These grey points around the information available by the manufacturers for the calculation of ABELIs could be that the companies consider device specifications as confidential because of some patent and trademarking considerations. That is why one of the recommendations of the study is to encourage the manufacturers to give not only the specific calculations and formulas to estimate ABELIs but also the filtering process before “raw data” is available for users. This may allow researchers and users to take decisions around how to interpret and compare data between companies´ equipment and device versions and software. Based on this limitation, we based our analysis in the available information already published in other high-quality peer review studies: a(t) [22,23], PL_RT_ [25], PL^TM^ [11], IL [26], PL_RE_ [17] and TL [28]; and the open documents of companies specifications.

## 5. Conclusions

Different conclusions could be extracted from this study: (1) Different accelerometry-based external load indexes used in sport science exist depending on the inertial device manufacturer company; (2) these ABELIs could vary due to the different algorithms and scaled values used during calculations, so it is not possible to compare the variables (e.g., different units and magnitudes, different sample frequencies); (3) due to that there were no differences between ABELIs when values are scaled and to the large to almost perfect relationship between ABELIs, both scaled and absolute values, all ABELIs seem to be reliable and sensible because all of them have the same origin—the accumulated change in acceleration of the three axes of movement.

In this sense, coaches and team staff should consider the following recommendations: (1) For accelerometer-based external workload comparison between players or teams, the same ABELIs need to be used, due to each ABELI having specific algorithms and scaled values during its calculations; and (2) even if the same formula is applied, the data between different models of the same company or between different companies cannot be compared for accelerometry-based workload due to different technical characteristics of the devices that could influence the final outcome.

Therefore, different solutions about the black-box data processing and calculation of ABELIs could be proposed: (1) a consensus with respect to the application of a universal ABELI for monitoring external workload by accelerometers; and (2) enabling their users to download the raw unprocessed accelerometry data; or (3) the availability and management of information regarding sampling rates, chip sets, filtering methods and data-processing algorithms in all devices. Thanks to this consensus, the variables of accelerometer-based workload monitoring can be compared among sports modalities, age categories and competition levels, among others, independently of the device used for this purpose. In addition, the recalculation of load indexes according to their preferences could be possible for players that are away with other teams, where the external load may have been captured with an accelerometer-device different from the one the club uses.

## Figures and Tables

**Table 1:** Descriptive data (mean ± SD; 95% CI in parentheses) of accelerometry-based external load indicators (ABELI) in young soccer players.

Title	Absolute		Relative	
ABELI	1st Period	2nd Period	1st Period	2nd Period
	Mean ± SD	Mean ± SD	Mean ± SD	Mean ± SD
	(95% CI, Lower to Upper)	(95% CI, Lower to Upper)	(95% CI, Lower to Upper)	(95% CI, Lower to Upper)
a(t) (*g*)	285,989.29 ± 4595.43	241,933.93 ± 73,977.35	6975.35 ± 113.47	5973.68 ± 1826.60
	(280,453.65 to 293,195.39)	(99,466.53 to 288,312.92)	(6924.78 to 7239.39)	(2455.96 to 7118.84)
PL_RT_ (a.u.)	58.17 ± 8.76	46.88 ± 14.99	1.44 ± 0.22	1.16 ± 0.37
	(43.06 to 73.43)	(21.17 to 65.46)	(1.06 to 1.81)	(0.52 to 1.62)
PL^TM^ (a.u.)	579.85 ± 86.65	467.2 ± 148.81	14.32 ± 2.14	11.54 ± 3.67
	(430.12 to 729.18)	(211.37 to 650.79)	(10.62 to 18.0)	(5.22 to 16.07)
IL (N)	29,162.65 ± 468.59	24,670.26 ± 7543.55	720.07 ± 11.57	609.14 ± 186.26
	(28,598.17 to 29,897.46)	(10,142.71 to 29,399.58)	(706.13 to 738.21)	(250.44 to 725.92)
PL_RE_ (a.u.)	556.51 ± 47.91	473.7 ± 152.98	13.74 ± 1.18	11.70 ± 3.78
	(493.38 to 651.76)	(197.16 to 635.77)	(12.18 to 16.09)	(4.87 to 15.70)
TL (a.u.)	57.98 ± 8.66	46.72 ± 14.88	1.43 ± 0.21	1.15 ± 0.37
	(43.01 to 72.92)	(21.13 to 65.08)	(1.06 to 1.80)	(0.52 to 1.61)

Note. a(t): Total acceleration (*g*); PL_RT_: Player Load by RealTrack Systems (a.u.); PL^TM^: PlayerLoad by Catapult Sports (a.u.); IL: Impulse Load (N); PL_RE_: Player Load by ZXY SporTracking (a.u.); TL: Total Load (a.u.).

**Table 2:** Agreement measurements of accelerometry-based external load indicators (ABELI) in young soccer players divided by match periods (absolute data).

Period	ABELI	Correlation			Comparison			
		*r* (*p* Value)	ICC	95% CI (L; U)	Bias	95% CI	*t* (*p* Value)	*d* (Rating)
1st	a(t) vs. PL^TM^	0.803 (<0.01)	0.03	−0.582; 0.621	285,409.4	−273,993.1; 844,811,9	199.41 (<0.01)	60.1 large
	a(t) vs. PL_RT_	0.805 (<0.01)	0.003	−0.6; 0.604	285,931.1	−274,493.9; 846,356.1	197.06 (<0.01)	59.4 large
	a(t) vs. IL	1 (<0.01)	0.202	−0.456; 0.717	256,826.6	−246,553.6; 760,206.8	196.79 (<0.01)	59.3 large
	a(t) vs. PL_RE_	0.958 (<0.01)	0.02	−0.589; 0.615	285,432.8	−274,015.5; 844,881	198.39 (<0.01)	59.8 large
	a(t) vs. TL	0.803 (<0.01)	0.665	0.105; 0.905	228,004.5	−218,884.4; 674,893.4	127 (<0.01)	38.29 large
	PL^TM^ vs. PL_RT_	1 (<0.01)	0.2	−0.457; 0.716	521.7	−500.8; 1544.2	21.18 (<0.01)	6.4 large
	PL^TM^ vs. IL	0.803 (<0.01)	0.287	−0.381; 0.758	−28,582.8	−84,605.1; 27,439.5	−224.66 (<0.01)	67.7 large
	PL^TM^ vs. PL_RE_	0.861 (<0.01)	0.729	0.227; 0.925	23.34	−22.4; 69.1	1.43 (0.186)	0.4 small
	PL^TM^ vs. TL	0.805 (<0.01)	0.02	−0.589; 0.615	−57,404.9	−169,918.5; 55,108.7	−21.16 (<0.01)	6.4 large
	PL_RT_ vs. IL	0.805 (<0.01)	0.03	−0.583; 0.621	−29,104.5	−86,149.3; 27,940.3	−199.4 (<0.01)	60.1 large
	PL_RT_ vs. PL_RE_	1 (<0.01)	0.306	−0.363; 0.767	-498.3	−1475.1; 478.4	−38.84 (<0.01)	11.7 large
	PL_RT_ vs. TL	0.958 (<0.01)	0.002	−0.601; 0.603	−579,226.6	−171,462.6; 55,609.5	−21.16 (<0.01)	6.4 large
	IL vs. PL_RE_	0.958 (<0.01)	0.194	−0.462; 0.713	28,608.1	−27,461.9; 84,674.2	213.91 (<0.01)	64.5 large
	IL vs. TL	0.803 (<0.01)	0.087	−0.544; 0.655	−28,822.1	−85,313.5; 27,669.2	−10.99 (<0.01)	3.3 large
	PL_RE_ vs. TL	0.861 (<0.01)	0.01	−0.596; 0.608	−57,428.2	−169,987.6; 55,131.1	−21.06 (<0.01)	6.3 large
2nd	a(t) vs. PL^TM^	0.919 (<0.01)	0.004	−0.6; 0.604	241,466.7	−231,808.1; 714,741.6	10.34 (<0.01)	3.1 large
	a(t) vs. PL_RT_	0.918 (<0.01)	0.001	−0.602; 0.602	241,887.1	−232,211.5; 715,985.7	10.34 (<0.01)	3.1 large
	a(t) vs. IL	1 (<0.01)	0.202	−0.456; 0.717	217,263.6	−208,573.1; 643,100.5	10.34 (<0.01)	3.1 large
	a(t) vs. PL_RE_	0.967 (<0.01)	0.004	−0.6; 0.605	241,460.2	−231,801.8; 714,722.3	10.34 (<0.01)	3.1 large
	a(t) vs. TL	0.919 (<0.01)	0.355	−0.314; 0.789	195,213.7	−187,405.1; 577,832.5	10.19 (<0.01)	3.1 large
	PL^TM^ vs. PL_RT_	1 (<0.01)	0.199	−0.458; 0.716	420.3	−403.5; 1244.2	9.93 (<0.01)	3 large
	PL^TM^ vs. IL	0.919 (<0.01)	0.036	−0.578; 0.625	−24,203.1	−71,641.1; 23,234.9	−10.33 (<0.01)	3.1 large
	PL^TM^ vs. PL_RE_	0.975 (<0.01)	0.974	0.901; 0.994	−6.5	−19.2; 6.2	−0.601 (0.563)	0.2 small
	PL^TM^ vs. TL	1 (<0.01)	0.02	−0.589; 0.615	−46,253.1	−136,909; 44,402.9	−9.93 (<0.01)	3 large
	PL_RT_ vs. IL	0.967 (<0.01)	0.004	−0.6; 0.604	−24,623.4	−72,885.2; 23,638.4	−10.34 (<0.01)	3.1 large
	PL_RT_ vs. PL_RE_	0.919 (<0.01)	0.189	−0.466; 0.71	−426.8	−1263.4; 409.8	−9.75 (<0.01)	2.9 large
	PL_RT_ vs. TL	0.967 (<0.01)	0.002	−0.601; 0.603	−46,673.4	−138,153.2; 44,806.4	−9.93 (<0.01)	3 large
	IL vs. PL_RE_	0.975 (<0.01)	0.039	−0.576; 0.626	24,196.6	−23,228.7; 71,621.8	10.35 (<0.01)	3.1 large
	IL vs. TL	0.918 (<0.01)	0.741	−0.252; 0.929	−22,050	−65,268; 21,168	−8.22 (<0.01)	2.5 large
	PL_RE_ vs. TL	1 (<0.01)	0.02	−0.589; 0.615	−46,246.5	−136,889.8; 44,396.7	−9.93 (<0.01)	3 large

Note. a(t): Total acceleration (*g*); PL_RT_: Player Load by RealTrack Systems (a.u.); PL^TM^: PlayerLoad by Catapult Sports (a.u.); IL: Impulse Load (N); PL_RE_: Player Load by ZXY SporTracking (a.u.); TL: Total Load (a.u.).

**Table 3:** Agreement measurements of accelerometry-based external load indicators (ABELI) in young soccer players divided by match periods (scaled and centered).

Period	ABELI	Correlation			Comparison			
		*r* (*p* Value)	ICC	95% CI (L; U)	Bias	95% CI	*t* (*p* Value)	*d* (Rating)
1st	a(t) vs. PL^TM^	0.803 (<0.01)	0.819	0.415; 0.952	0	0	0 (1)	0, trivial
	a(t) vs. PL_RT_	0.805 (<0.01)	0.821	0.42; 0.953	0	0	0 (1)	0, trivial
	a(t) vs. IL	1 (<0.01)	1	1; 1	0	0	0 (1)	0, trivial
	a(t) vs. PL_RE_	0.958 (<0.01)	0.962	0.856; 0.991	0	0	0 (1)	0, trivial
	a(t) vs. TL	0.803 (<0.01)	0.819	0.415; 0.952	0	0	0 (1)	0, trivial
	PL^TM^ vs. PL_RT_	1 (<0.01)	1	1; 1	0	0	0 (1)	0, trivial
	PL^TM^ vs. IL	0.803 (<0.01)	0.819	0.415; 0.952	0	0	0 (1)	0, trivial
	PL^TM^ vs. PL_RE_	0.861 (<0.01)	0.873	0.564; 0.967	0	0	0 (1)	0, trivial
	PL^TM^ vs. TL	0.805 (<0.01)	0.861	0.538; 0.964	0	0	0 (1)	0, trivial
	PL_RT_ vs. IL	0.805 (<0.01)	1	1; 1	0	0	0 (1)	0, trivial
	PL_RT_ vs. PL_RE_	1 (<0.01)	0.865	0.548; 0.965	0	0	0 (1)	0, trivial
	PL_RT_ vs. TL	0.958 (<0.01)	1	1; 1	0	0	0 (1)	0, trivial
	IL vs. PL_RE_	0.958 (<0.01)	0.958	0.842; 0.99	0	0	0 (1)	0, trivial
	IL vs. TL	0.803 (<0.01)	0.803	0.39; 0.947	0	0	0 (1)	0, trivial
	PL_RE_ vs. TL	0.861 (<0.01)	0.861	0.538; 0.964	0	0	0 (1)	0, trivial
2nd	a(t) vs. PL^TM^	0.919 (<0.01)	0.919	0.71; 0.979	0	0	0 (1)	0, trivial
	a(t) vs. PL_RT_	0.918 (<0.01)	0.918	0.707; 0.979	0	0	0 (1)	0, trivial
	a(t) vs. IL	1 (<0.01)	1	1; 1	0	0	0 (1)	0, trivial
	a(t) vs. PL_RE_	0.967 (<0.01)	0.967	0.874; 0.992	0	0	0 (1)	0, trivial
	a(t) vs. TL	0.919 (<0.01)	0.919	0.71; 0.979	0	0	0 (1)	0, trivial
	PL^TM^ vs. PL_RT_	1 (<0.01)	1	1; 1	0	0	0 (1)	0, trivial
	PL^TM^ vs. IL	0.919 (<0.01)	0.919	0.71; 0.979	0	0	0 (1)	0, trivial
	PL^TM^ vs. PL_RE_	0.975 (<0.01)	0.975	0.902; 0.994	0	0	0 (1)	0, trivial
	PL^TM^ vs. TL	1 (<0.01)	1	1; 1	0	0	0 (1)	0, trivial
	PL_RT_ vs. IL	0.967 (<0.01)	0.918	0.707; 0.979	0	0	0 (1)	0, trivial
	PL_RT_ vs. PL_RE_	0.919 (<0.01)	0.974	0.898; 0.993	0	0	0 (1)	0, trivial
	PL_RT_ vs. TL	0.967 (<0.01)	1	1; 1	0	0	0 (1)	0, trivial
	IL vs. PL_RE_	0.975 (<0.01)	0.967	0.874; 0.992	0	0	0 (1)	0, trivial
	IL vs. TL	0.918 (<0.01)	0.919	0.71; 0.979	0	0	0 (1)	0, trivial
	PL_RE_ vs. TL	1 (<0.01)	0.975	0.902; 0.994	0	0	0 (1)	0, trivial

Note. a(t): Total acceleration (*g*); PL_RT_: Player Load by RealTrack Systems (a.u.); PL^TM^: PlayerLoad by Catapult Sports (a.u.); IL: Impulse Load (N); PL_RE_: Player Load by ZXY SporTracking (a.u.); TL: Total Load (a.u.).
